# Pharmacological Activities of Safflower Yellow and Its Clinical Applications

**DOI:** 10.1155/2022/2108557

**Published:** 2022-06-27

**Authors:** Yan Chen, Meifeng Li, Jiayu Wen, Xiaoqi Pan, Zixin Deng, Junren Chen, Guanru Chen, Lei Yu, Yunli Tang, Gangmin Li, Xiaofang Xie, Cheng Peng

**Affiliations:** ^1^State Key Laboratory of Southwestern Chinese Medicine Resources, Chengdu 611137, China; ^2^School of Public Health, Chengdu University of Traditional Chinese Medicine, Chengdu 611137, China; ^3^College of Pharmacy, Chengdu University of Traditional Chinese Medicine, Chengdu 611137, China; ^4^South China Branch of National Engineering Research Center for Manufacturing Technology of Solid Preparation of Traditional Chinese Medicine, Guangxi University of Traditional Chinese Medicine, Nanning, Guangxi 530200, China

## Abstract

**Background:**

Safflower is an annual herb used in traditional Chinese herbal medicine. It consists of the dried flowers of the Compositae plant safflower. It is found in the central inland areas of Asia and is widely cultivated throughout the country. Its resistance to cold weather and droughts and its tolerance and adaptability to salts and alkalis are strong. Safflower has the effect of activating blood circulation, dispersing blood stasis, and relieving pain. A natural pigment named safflower yellow (SY) can be extracted from safflower petals. Chemically, SY is a water-soluble flavonoid and the main active ingredient of safflower. The main chemical constituents, pharmacological properties, and clinical applications of SY are reviewed in this paper, thereby providing a reference for the use of safflower in preventing and treating human diseases.

**Methods:**

The literature published in recent years was reviewed, and the main chemical components of SY were identified based on chemical formula and structure. The pharmacological properties of hydroxysafflor yellow A (HSYA), SYA, SYB, and anhydrosafflor yellow B (AHSYB) were reviewed.

**Results:**

The main chemical constituents of SY included HSYA, SYA, SYB, and AHSYB. These ingredients have a wide range of pharmacological activities. SY has protective effects on the heart, kidneys, liver, nerves, lungs, and brain. Moreover, its effects include, but are not limited to, improving cardiovascular and cerebrovascular diseases, abirritation, regulating lipids, and treating cancer and diabetic complications. HSYA is widely recognised as an effective ingredient to treat cardiovascular and cerebrovascular diseases.

**Conclusion:**

SY has a wide range of pharmacological activities, among which improving cardiovascular and cerebrovascular diseases are the most significant.

## 1. Introduction

Safflower is native to Central Asia and is a traditional Chinese herb that consists of the dried flowers of the Compositae plant safflower [1]. The *Compendium of Materia Medica* states that safflower has the effect of “blood circulation, moisturising the skin, analgesic effect, reducing swelling, reducing menstrual bleeding, and improving blood stasis to eliminate edema” [2]. As a traditional medicine, safflower is often used to relieve pain, fight inflammation, and improve micro-circulation in China, the Middle East, and other countries [3].

Safflower yellow (SY) is extracted from safflower petals. It is a water-soluble flavonoid and the main active ingredient of safflower [4]. There are at least 29 compounds in SY (Table 1) [5], and the main compounds include hydroxysafflor yellow A (HSYA), anhydrosafflor yellow B (AHSYB), and other small-molecule chemicals. HSYA, especially, plays an important pharmacological role [6, 7]. Messelli et al. [8] isolated HSYA from safflower for the first time. Owing to its abundance and strong biological activity, HSYA is recognised in the *Chinese Pharmacopoeia* as one of the standard ingredients for the quality control of safflower.

Modern pharmacological studies show that SY has several pharmacological activities, which include but are not limited to improving cardiovascular and cerebrovascular diseases, abirritation [9], regulating lipids [10], and treating cancer [11] and diabetic complications [12]. SY is approved by the Food and Drug Administration of China. Safflower plays an important role in the treatment of cardiovascular and cerebrovascular diseases [13]. The biological activity of SY has been studied conventionally in vitro and in vivo using an experimental mouse model. SY has been reported to have positive effects on the heart, brain, and kidneys of mice. The disease-resistant components of SY and their mechanisms of action are shown in Table 2.

**Figure 1:**
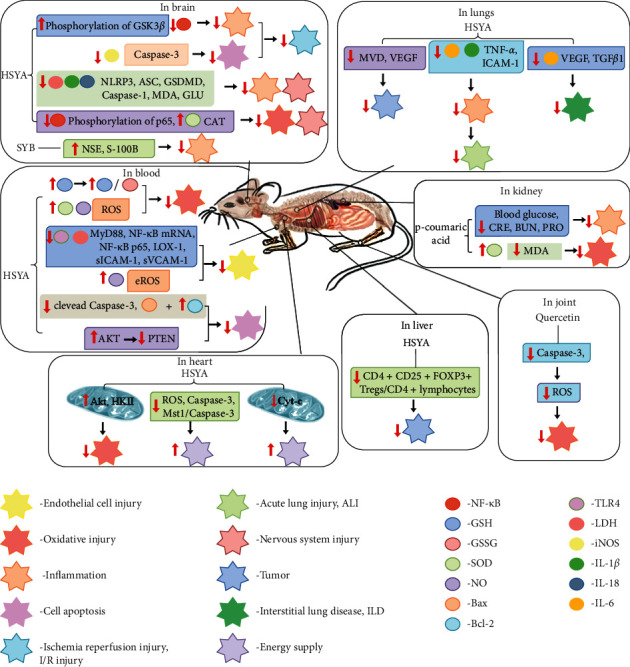
The major biological activities of SY.

In this study, research progress on the main chemical constituents, pharmacological activities of SY (Figure 1), and its clinical applications (Table 3) were reviewed and summarised, providing a reference for the further development and utilisation of safflower.

## 2. Pharmacological Activities

### 2.1. Improving Cardiovascular and Cerebrovascular Diseases (CCVDs)

Several in vivo and in vitro studies have shown that SY has significant effects in alleviating CCVDs, including the prevention of atherosclerosis and thrombosis, and as a cardioprotective and neuroprotective. Moreover, the closely related anti-oxidant [39], anti-inflammatory [40], and neuroprotective effects [41] play a role in the prevention and treatment of CCVDs.

#### 2.1.1. Prevention and Treatment of Atherosclerosis

Atherosclerosis is the main cause of cardiovascular and cerebrovascular diseases. This condition mainly occurs in vascular endothelial cells of the large and medium arteries, which are monolayer cells located between plasma and vascular tissues. Endothelial cell injury is considered the basic pathological condition in atherosclerosis. SY is a promising natural product for the treatment of atherosclerosis [42].

Human umbilical vein endothelial cells (HUVECs) are often used as an in vitro model to study the role of endothelial cells. Xie et al. [19] established a hydrogen peroxide (H_2_O_2_) induced HUVEC model of oxidative damage and found that HSYA could alleviate the H_2_O_2_-induced oxidative damage in HUVECs through upregulating glutathione (GSH) levels, decreasing intracellular reactive oxygen species (ROS) levels, increasing the expression of AKT and Bcl-2 proteins, and inhibiting the expression of Bax and PTEN proteins. The mechanism may be related to the expression of the Bax/Bcl-2 and AKT/PTEN signalling pathways. HSYA significantly upregulates the proliferation rate and downregulates the mRNA and protein expression of TLR4, MyD88, and nuclear transcription factor-*κ*B (NF-*κ*B) mRNA in a dose-dependent manner in vitro in lipopolysaccharide (LPS) induced endothelial injury in HUVECs and significantly downregulates NF-*κ*B p65 expression [43].

Cui et al. [44] established an H_2_O_2_-induced oxidative stress injury model of human vascular endothelial cells EC-304 and found that HSYA could significantly improve the cell survival rate, enhance intracellular superoxide dismutase (SOD) activity, increase intracellular NO levels, and enhance Bcl-2 expression in a dose-dependent manner. It could also reduce the expression of Bax, caspase-3, and cleaved caspase-3. Moreover, HSYA plays a protective role in high glucose-induced vascular injury by inhibiting the generation of H_2_O_2_ and ROS, activation of NADPH oxidase 4, and the adhesion of adhesion molecules and monocyte endothelial cells [45]. Miao et al. [20] established an oxidised low-density lipoprotein (OX-LDL) induced human coronary artery endothelial cell (HCAEC) model of injury to explore the protective effects of HSYA. The effect was mainly through upregulating the expression of endothelial nitric oxide (NO) synthase (eNOS) gene and protein and increasing NO release. By downregulating the lectin-like low-density lipoprotein receptor 1 (LOX-1) mRNA and protein expression and inhibiting lactate dehydrogenase (LDH) release, HSYA could inhibit the damage induced by high OX-LDL levels in HCAECs, protect cells, and promote cell repair.

**Figure 2:**
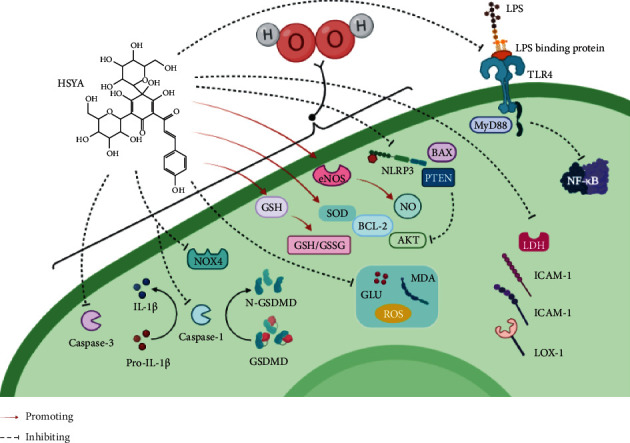
The mechanism of HSYA protecting and repairing endothelial cell injury.

HSYA can also affect the adhesion function of vascular endothelial cells, significantly reduce soluble intercellular adhesion molecule (ICAM)-1 and soluble vascular cell adhesion molecule (VCAM)-1 levels in the serum of male Sprague-Dawley (SD) rats, and inhibit the expression of VCAM-1 and ICAM-1 on the surface of the thoracic aorta [46]. The biological mechanism is shown in Figure 2.

#### 2.1.2. Anti-Thrombotic Effects

SY can promote blood circulation and remove blood stasis [40]. Adenosine diphosphate (ADP) is the main component leading to platelet aggregation. SY may affect the expression of activated glycoproteins on platelet membranes by affecting the activation of the downstream conductor of ADP receptors, thereby inhibiting ADP-induced platelet aggregation in humans. The inhibition of platelet aggregation is mainly manifested by the following aspects: ADP receptor transduction and expression of PAC-1 glycoprotein on platelet membranes, calcium ion activation, and regulation of the levels of cyclic adenosine monophosphate, arachidonic acid, and thromboxane (TX) A_2_ in intracellular platelets by ADP [47]. Wang et al. [48] established a phenylhydrazine-induced thrombosis model and verified that HSYA could significantly inhibit thrombosis in vivo and protect the body from exogenous or disease-induced endogenous toxins by promoting blood circulation and accelerating toxin excretion. Studies have investigated the effects of intravenous HSYA injections on the rate of dissolution of blood clots, blood fibrinogen (FIB) levels, prothrombin time (PT), blood coagulation time in experimental animals, and thrombus formation in vivo and in vitro. HSYA has significant thrombolytic effects and can reduce the FIB content to inhibit platelet aggregation and prolong clotting time and PT in rats [49]. SYE not only can inhibit thrombosis in the cerebral arterioles of mice but also has a protective effect against haemorrhagic disorders in rats with blood stasis syndrome [5].

**Figure 3:**
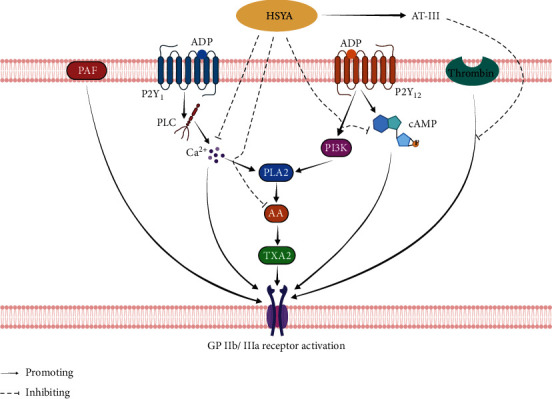
The mechanism of HSYA inhibiting platelet aggregation.

HSYA is used in a clinical setting owing to its anti-coagulant effects [50]. The combination of HSYA and low-molecular-weight heparin calcium has a synergistic anti-coagulant effect, which can reduce the incidence of lower limb venous thrombosis [51]. The relevant mechanisms are as follows: (1) SY can antagonise the platelet-activating factor (PAF) receptors and indirectly inhibit platelet aggregation (PAF is an important factor contributing to platelet aggregation, serotonin release, and increasing free Ca^2+^ concentration in platelets) [52]; (2) intravenous injection of SY can reduce the activity of inhibitor plasminogen activator and significantly improve the activity of tissue plasminogen activator in plasma; and (3) SY can combine with hydroxyl free radicals to form HSYA and enhance the ability of endothelial cells to release AT-III, inhibit thrombin activity, and, thus, enhance the anti-thrombosis effect. The biological mechanism is shown in Figure 3.

#### 2.1.3. Myocardial Protection

Myocardial ischemia, occlusion of blood-supplying arteries, and metabolic disorders can lead to myocardial hypoxia. Reperfusion caused by tissue damage is usually aggravating and can lead to myocardial ischemia/reperfusion (I/R) injury. Myocardial I/R injury poses a serious threat to human health and is one of the important risk factors for myocardial infarction (MI) associated with several complex pathological changes.

SY shows potential in treating myocardial I/R and might protect from myocardial ischemia by reducing myocardial oxygen consumption. It could improve the ability of normal rats to resist hypoxia at normal pressure and prolong the survival time of rats under hypoxia, suggesting the effectiveness of SY pigment [53]. Studies have reported that the expression of the anti-apoptotic protein Bcl-2 decreased and that of the proapoptotic protein Bax increased in hypoxia/reoxygenation (A/R) injured primary myocardial cells of suckling rats, whereas these trends were reversed after HSYA treatment. The anti-apoptotic effect of HSYA is lost after the addition of the phosphatidylinositol-3-kinase (PI3K) inhibitor LY294002, which indicates that HSYA can reduce apoptosis in A/R-injured myocardial cells by activating the PI3K pathway [54]. HSYA can also protect the cardiomyocytes of rats from A/R injury by regulating the phosphatidylinositol-3-kinase/protein kinase B/glycogen synthase kinase 3*β* (PI3K/Akt/GSK3*β*) signalling pathway.

HSYA can reduce serum angiotensin II (Ang II), TXB_2_, and LDH levels in the cardiovascular system and protect endothelial cells and preserve myocardial systolic function. These findings indicate that HSYA provides cardiac protection by increasing myocardial blood flow and oxygen supply to effectively alleviate heart disease caused by myocardial ischemia and other ischemic factors [55].

In both the mouse model of MI and the SD rat model of I/R, HSYA can reduce the area of MI and alleviate the deterioration in cardiac function after MI to varying degrees, specifically by reducing myocardial cell apoptosis and myocardial fibrosis [56, 57]. In addition, HSYA can improve autophagy and inhibit the NLRP3 inflammasome [58] by inhibiting the mTOR pathway and activating AMPK.

#### 2.1.4. Brain Protection

HSYA is widely used to treat cerebrovascular diseases and in the protective treatment of I/R injury [59]. The proteomic analysis reported by Xu et al. [60] showed that the mTOR, Eftud2, Rab11, Ppp2r5e, and HIF-1 signalling pathways are key central proteins and important pathways of HSYA in preventing cerebral I/R injury. HSYA can reduce cerebral infarction volume; decrease the neurological deficit score; increase GSK3*β* phosphorylation; inhibit the activation of iNOS, NF-*κ*B, and caspase-3; and decrease iNOS, NF-*κ*B, and caspase-3 activity in the penumbra after cerebral I/R. HSYA exerts an anti-inflammatory and anti-apoptotic effect by regulating GSK-3*β* phosphorylation, thereby reducing I/R injury [61]. Cao et al. [62] established in vitro oxygen-glucose deprivation (OGD) model using brain micro-vascular endothelial cells (BMECs) and evaluated the protective effects of astragaloside IV (AS-IV) and HSYA. Their results showed that AS-IV and HSYA significantly attenuated OGD-induced cell loss by increasing cell proliferation and inhibiting apoptosis, and HSYA treatment protected bone marrow mesenchymal stem cells from IR injury by stimulating vascular endothelial growth factor and NOS signalling. AS-IV and HSYA show synergistic effects in the in vitro rescue of BMECs by downregulating PHLPP-1 expression and activating the Akt signalling pathway. HSYA combined with the blood-brain barrier (BBB) modulator Lex can significantly reduce the volume of cerebral infarction, improve histopathological morphology, recruit brain-derived neurotrophic factors, and alleviate cerebral I/R injury [21].

The neuroprotective effect of HSYA on focal cerebral ischemia is mainly achieved by regulating the crosstalk between Janus kinase (JAK) 2/signal transducer and activator of transcription (STAT) 3 and the suppressors of cytokine signalling (SOCS) 3 pathway. Its effect can be speculated as follows: HSYA may inhibit JAK2-mediated signal transduction, further activate p-JAK2/p-STAT3 expression, and then stimulate the downstream activation of SOCS3, resulting in the negative feedback signal by SOCS3 to p-JAK2/p-STAT3. It is also possible that HSYA can directly activate SOCS3, thereby neutralising JAK2/STAT3 activation, which is detrimental [63]. HSYA can downregulate the expression of cytokines, including NLRP3, ASC, caspase-1, gasdermin D, interleukin (IL)-1*β*, IL-18, LDH, NF-*κ*B, and p-p56, suggesting its inhibition of the activation of cellular scortosis and apoptotic pathways during nerve injury [21].

HSYA can enhance the expression of epidermal growth factor receptor, hypoxia-inducible factor 1*α*, and eNOS and promote angiogenesis [64]. Among them, eNOS activation can alleviate neurovascular injury and improve functional prognosis after a stroke, and HSYA can upregulate eNOS levels [65]. HSYA can reduce malondialdehyde levels and increase glutathione and SOD levels to inhibit ROS and can also activate Akt and *β*-catenin signals to promote nerve cell survival [66].

#### 2.1.5. Anti-Oxidant Effects

SY exerts significant anti-oxidant effects by removing hydroxyl free radicals and inhibiting lipid peroxidation [67]. HSYA and SYB are the two major active substances in SY that contribute to this effect. HSYA can directly remove hydroxyl free radicals in a dose-dependent manner, whereas SYB inhibits Fenton's oxidative damage to 2-deoxyribose. The anti-oxidant effects of both these components are potent [37, 68–70].

SOD level decreases in lens epithelial cells undergoing oxidative damage. HSYA can significantly increase SOD levels to protect against lens epithelial cell damage [71]. Similarly, HSYA can increase the activities of SOD and catalase in brain-injured rats with trauma and can also reduce malondialdehyde and glutathione levels in acute ischemic stroke [72].

HSYA can reduce the expression of the metabolite 15-hydroxyeicosatetraenoic acid (HETE) in a dose-dependent manner and reduce the breakdown of the BBB, indicating its role in reducing oxidative stress, inhibiting 12/15-lipoxygenase activity, and protecting the relative integrity, structure, and function of the BBB, thereby exerting a brain-protective action [73]. HSYA can also downregulate micro-RNA-1 expression and ROS release in H9c2 cells subjected to oxidative injury [74].

NO has various physiological functions. In the vascular system, NO is mainly released by endothelial cells to prevent their apoptosis. H_2_O_2_ is commonly used to induce oxidative stress injury in human umbilical vein vascular endothelial cell lines (EC-304) [75–77], and HSYA treatment can effectively increase SOD activity and NO content [44, 78]. In conclusion, HSYA exerts anti-oxidant effects by regulating the activity or level of related enzymes.

#### 2.1.6. Anti-Inflammatory Effects

SY has an anti-inflammatory effect [79, 80]. It can downregulate the expression of toll-like receptor 4 (TLR4) and NF-*κ*B p65 proteins in myocardial tissue of rats after myocardial I/R injury and significantly reduce serum tumour necrosis factor *α* (TNF-*α*) and IL-6 levels, indicating that SY plays an effective inhibitory role in affecting the inflammatory response to myocardial I/R injury in rats. Its mechanism might be related to regulating the TLR-NF-*κ*B pathway [81, 82]. Besides, the anti-inflammatory mechanism of SY might also be related to the inhibition of the MAPK pathway. It has been reported that SY injection can inhibit p38 MAPK phosphorylation and NF-*κ*B activation in a dose-dependent manner, resulting in a reduction in the levels of TNF-*α*, IL-1*β*, IL-6, ICAM-1, VCAM-1, and adhesion molecules in a rat model of oleic acid-induced acute lung injury [83, 84].

HSYA can improve airway function and relieve inflammation in patients with bronchial asthma by inhibiting immunoglobulin E and platelet-activating factor (PAF) levels, rebalancing T helper 1 (Th1)/Th2 cells, and blocking the MAPK signalling pathway. These findings suggest that HSYA plays a multifunctional role in the prevention and treatment of asthma. Due to the multitarget characteristics of HSYA and its few side effects, it is being used in the research and development of new anti-asthma drugs [85].

IL-10 is an anti-inflammatory factor that can inhibit the synthesis and secretion of proinflammatory factors during an inflammatory response. However, excessive IL-10 can cause nonspecific immune disorders in the body. SY pigment can inhibit the excessive increase in IL-10 and reduce TNF-*α* and IL-6 levels to reduce inflammation due to viper bite poisoning [86]. In summary, HSYA can inhibit signal transduction and the expression of inflammatory factors to reduce the inflammatory response; moreover, it has a certain anti-inflammatory effect.

### 2.2. Abirritation

HSYA has a strong analgesic effect that might be related to the inhibition of the MAPK/p38/iNOS pathway and NO release. HSYA can significantly reduce writhing in rats following treatment with acetic acid and can also increase their pain threshold (pain induced by a hot plate) in a dose-dependent manner. HSYA can significantly reduce the NO content of LPS-induced macrophage RAW264.7 cells by inhibiting the MAPK/p38/iNOS signalling pathway [26].

HSYA injections have been recently reported to be effective as an adjuvant drug in the management of angina pectoris [9, 87, 88]. The proposed mechanisms of action include regulating the intracellular flow of Ca^2+^, promoting blood circulation, vasodilation, and increasing blood supply for tissue and organs. High blood viscosity can cause angina pectoris. Fortunately, the SY pigment can release prostacyclin by activating vascular endothelial cells and increasing coronary blood flow [89, 90]. These reported findings indicate that HSYA can activate vascular endothelial cells to release prostaglandins by inhibiting the MAPK/P38/iNOS pathway, thus resulting in an analgesic effect.

### 2.3. Lipid Regulation

SY can lower blood lipids [91, 92]. Intravenous injections of SY can significantly reduce serum low-density lipoprotein cholesterol (LDL-C), triglyceride (TG), and total cholesterol (TC) levels in rats with hyperlipidaemia and increase high-density lipoprotein cholesterol levels [49].

HSYA can also improve the pathological morphology of organs caused by hyperlipidaemia. The liver plays an important role in lipid metabolism and the synthesis of endogenous cholesterol. Dyslipidaemia can easily increase the burden on the liver leading to hepatic dysfunction. Chai et al. established a fatty liver model of hyperlipidaemia in rats [93]; when treated with HSYA, the lipid droplets in liver cells reduced remarkably in a dose-dependent manner.

Li et al. found that HSYA could significantly reduce the mRNA expression of fatty acid synthase, peroxisome proliferator-activated receptor (PPAR-*γ*), and stearoyl-coadesaturase1 in liver tissues. Silent information regulator 1 (Sirt1) is a histone deacetylase that can regulate gene expression by regulating gene transcription. Sirt1 can inhibit the expression and activity of PPAR-*γ* and downregulate the expression of fat storage-related genes. HSYA can protect the liver by affecting Sirt1 to regulate the high fat-induced lipid accumulation in the liver [34]. HSYA can regulate receptor expression and lower LDL levels, thereby lowering blood lipid levels.

Yin et al. administered intramuscular vitamin D3 injections and nicotine gavage to establish a rat model of atherosclerosis. They found that HSYA could reduce serum lipid levels, but it was not better than the classic lipid-lowering drug simvastatin at a human equivalent dose [92, 94]. Therefore, HSYA can be used as an adjuvant in lipid-lowering therapy [95].

### 2.4. Anti-Cancer Effects

Within a certain dose range, HSYA can inhibit several carcinogens by inhibiting tumour neovascularisation, also inhibit the growth of several types of tumour cells, and induce tumour cell apoptosis [96].

Tumour angiogenesis is an important factor leading to tumour growth, invasion, and metastasis [97]. It has been reported that 28 mg/L of HSYA can effectively inhibit the growth of transplanted tumour tissues in rats, indicating that HSYA has an inhibitory effect on tumour angiogenesis [98].

MDA-MB-231 is a malignant, invasive, triple-negative breast cancer cell line that is resistant to certain chemotherapy drugs [99]. MDA-MB-231 is the ideal in vitro model to study drugs used to treat breast cancer [100]. HSYA can inhibit the metastasis-related protein matrix metalloproteinase 2 (MMP2) in MDA-MB-231 cells to inhibit the migration and invasion of breast cancer cells and can promote apoptosis of breast cancer cells by activating the caspase-3-dependent apoptosis pathway [35]. In addition, studies have been performed to screen the in vitro activity of SY by using time- and dose-dependent cell response spectra induced by epidermal growth factor (EGF) and to evaluate the anti-metastasis effect of SY by orthotopic pulmonary metastasis and intravenous injection. The results show that SY inhibits the EGF-mediated time and dose-dependent cell response spectra by inhibiting cytoskeletal rearrangement. Moreover, SY has significant inhibitory effects on cell migration in vitro and lung metastasis of breast cancer cells in vivo. Consistent with these phenotypes, in SY-treated MDA-MB-231 cells and in lung metastases, EGF stimulation reduces invasion-site formation and MMP-9 and P-SRC protein expression. These data suggest that the anti-metastasis effect of SY is due to its inhibition of invasive cytoskeleton formation, which is mainly mediated by P-SRC protein [101]. In conclusion, SY can inhibit MMP2, caspase-3, and P-SRC in MDA-MB-231 cells to inhibit breast cancer and exert an anti-metastatic effect.

Ovarian cancer has the highest mortality rate among gynaecological malignancies. Cisplatin-based chemotherapy is the basic postoperative treatment [102]. Multiple studies have reported that protein kinase B (PKB, also known as Akt) activators can promote chemoresistance and increase the survival of ovarian cancer cells by attenuating the p53 proapoptotic signal [103]. Yang et al. found that phosphatidylinositide 3-kinase/protein kinase B (PI3K/AKT) has a significant effect on cisplatin resistance in human epithelial ovarian cancer, and the inhibition of the PI3K/AKT pathway can significantly increase the sensitivity of tumour cells to cisplatin [104]. Liang et al. transplanted the cisplatin-resistant human ovarian cancer cell line A2780/DDP into nude rats to study drug intervention and found that HSYA enhanced the sensitivity of A2780/DDP to cisplatin by downregulating the PI3K/AKT pathway and inhibiting the p-AKT protein [105]. The results suggest that HSYA can reduce drug resistance in ovarian cancer.

Earlier, Wang et al. applied HSYA directly to BG C823 human gastric cancer cells and found that it significantly inhibited the proliferation of BG C823 cells after 48 h [106].

### 2.5. Treatment of Diabetic Complications

Diabetic nephropathy (DN) is one of the major micro-vascular complications of diabetes mellitus (DM) disability and death [12, 107]. SY can prolong PT. HSYA is a platelet-activating factor receptor antagonist that can decrease 5-hydroxytryptophan and free Ca^2+^ concentration in platelets and cause platelet adhesion. As a result, HSYA can reduce blood glucose levels, blood viscosity, and homeostasis model assessment in patients with early type 2 DN to relieve renal damage and improve insulin resistance [108].

In DM, long-term high glucose levels can induce excessive ROS leading to apoptosis and functional damage to islet *β* cells. In such cases, HSYA can reduce excessive ROS production by inhibiting the JNK/c-jun signalling pathway, thereby significantly protecting *β* cells and islet function [109].

## 3. Pharmacokinetic Studies

HSYA is a representative chemical compound of the biopharmaceutics classification system class III drugs. Pharmacokinetic studies show that HSYA has high water solubility but poor intestinal membrane permeability, resulting in low oral bioavailability of only 1.2%, of which 48% of the prototype drug is excreted in the urine, 2.9% in faeces, and only 0.062% ± 0.011% in the bile. Similarly, 88.6% was directly excreted in the urine after intravenous administration [40, 110]. In addition, owing to its polarity and solubility, HSYA is easily degraded and metabolised in the liver and gastrointestinal tract, leading to malabsorption. Moreover, it is eliminated quickly and has a very short half-life [111]. Safflower oral solution and SY injection are HSYA-containing preparations currently used in clinical practice [112]. Disease status can affect the metabolism of SY pigments in the body. Yao et al. [113] first reported the pharmacokinetic differences in HSYA between normal mice and streptozotocin-induced mice with dilated cardiomyopathy. Mice in the DCM group exhibited a significantly higher area under the curve (AUC0-t and AUC0-∞) and peak plasma concentration for HSYA than those in the normal group, suggesting a high uptake of HSYA by mice in the DCM group. Furthermore, the plasma clearance and apparent volume of distribution were significantly lower in mice in the DCM group than those in the HSYA-treated group, indicating slower elimination in the DCM group than in the normal group. These results indicated significant changes in HSYA pharmacokinetics in mice with diseases.

HSYA is distributed in the heart, liver, spleen, lungs, kidneys, brain, and gastrointestinal tract of rats after the oral administration of safflower water extract and SY pigment, but the content was higher only in the gastrointestinal tract and lungs [114]. After intravenous safflower injection, the AUC of HSYA in different organs was in the order of blood, kidneys, liver, lungs, heart, and spleen but was not detected in the brain, possibly because HSYA could not easily cross the BBB [115]. However, relevant studies have shown upon injection; HSYA can enter the brain of rats with traumatic brain injury and play a protective role in the nervous system [72].

Jin et al. [116] first conducted a comparative study between the metabolism in normal rats and in those with blood stasis syndrome after the intragastric administration of HSYA. In addition to the prototype drug, eight related metabolites were detected in normal rats, including five phase I metabolites (hydrolysis, reduction, hydroxylation, hydration, and methylation) and three phase II metabolites (acetylation, gluconal acidification, and gluconal acidification plus hydroxylation). However, only seven metabolites were detected in rats with blood stasis syndrome, and no glucoaldehyde acidification and hydroxylation products were detected, indicating that the metabolites of HSYA differed among animal models. Moreover, the hydroxylated, hydroxylated plus methylated, acetylated, and glucoaldehyde-acidified metabolites were higher among other metabolites, suggesting their role in the pharmacological activity of HSYA [117].

Besides, related research has found that after rats were administered safflower water extract and SY pigment, the cumulative excretion rate of HSYA was the highest, followed by that in the urine, and the least in the bile. HSYA is mainly excreted in the urine during intravenous administration [114]. However, Jia et al. [118] found that when rats were intravenously administered Xuebijing injection, the urine:faeces drug-excretion ratio reached the maximum value 8–12 h after administration; approximately 50% of HSYA was detected in the stools and 3.41% in the urine after 25 h, suggesting that HSYA was mainly excreted from stools after intravenous administration, which may be related to other components in Xuebixin.

## 4. Clinical Applications of SY Pigment

SY is a valuable natural pigment and the main active ingredient of safflower. SY has multiple positive traits, including its bright colour; resistance to high and low temperature, high pressure, light, acid, and reducing agent; and anti-microbial properties. SY has long been used in a clinical setting.

In China, safflower has long been used as an important herbal medicine, with the first known record in the Song Dynasty in “Kai Bao Ben Cao.” Safflower has been cultivated for more than 2,000 years. It has the effects of activating blood to regulate menstruation and resolving blood stasis to relieve pain. It is compatible with other drugs traditionally used to treat amenorrhea, menorrhagia, lochia, stasis, masses in the body, chest pain, abdominal pain, trauma and fracture, swelling, and ulcers on the body surface. Safflower is currently used both internally and externally to treat gynaecological and obstetrical conditions, cardiovascular and cerebrovascular diseases, trauma, and fractures (Table 3) [119, 120].

The chemical constituents of safflower are complex and include flavonoids, steroids, phenolic acids, diols, lignans, chalcone, alkynes, and volatile oils. Among them, SY is the major active ingredient. HSYA is the key active ingredient in SY. Therefore, HSYA is usually used as an indicator for the quality control of SY as described in pharmacopoeia [121]. Owing to their diverse pharmacological effects, low costs, and other advantageous traits, herbal compounds are increasingly attracting the attention of scientists worldwide [122]. AHSYB, the second-most prominent ingredient in SY after HSYA, contains phenolic hydroxyl groups that are responsible for its anti-oxidant effect. AHSYB has a good dyeing effect, making it a useful colouring agent for tablets and in preparing sugar coatings [123].

Safflower is used in traditional Chinese medicine for promoting blood circulation and removing blood stasis. It has a definite curative effect on cardiovascular and cerebrovascular diseases and has broad application prospects. HSYA can be used as a vascular relaxant and shows potential in the treatment of cardiovascular diseases [124]. TRPV4 is related to vascular tension. Yang et al. [124] found that HSYA can increase Ca^2+^ levels in endothelial cells through the TRPV4 channel and activate eNOS and its phosphorylation through protein kinase A, thus promoting NO production and leading to vascular relaxation. HSYA causes vasodilation mainly by the activation of the BKCa channel, inhibition of the L-type calcium channel, and reduction of intracellular free Ca^2+^ levels [125]. As a Chinese herbal medicine, SY, the main active ingredient, can be used in the treatment of myocardial I/R injury. Using in vivo and in vitro models, Lu et al. [4] studied whether SY could reduce myocardial I/R in rats and provided the theoretical basis for its use as a potential drug to treat myocardial I/R injury. In vivo experiments show that safflower can improve cardiac function after myocardial I/R injury and effectively attenuate I/R-induced MI. N-acetylcysteine can be used to remove ROS in an in vivo I/R model, or a preinjection of SY into the internal jugular vein of rats can reduce the expression of the inflammatory cytokines IL-6 and TNF-*α*, especially IL-1*β*. An in vitro myocardial model shows that SY reduces I/R injury by inhibiting the release of LDH and reactive ROS. In vitro studies have further verified and explained the potential mechanism of its cardioprotective effects. In addition, SY pretreatment significantly reduced I/R-induced NLRP3 expression and caspase-1 activation. In summary, these results suggest that SY intervention before reperfusion can reduce myocardial I/R injury. However, the protective mechanism of SY in myocardial I/R injury needs further study and experimental analysis.

In 2017, the World Health Organization reported that chronic obstructive pulmonary disease (COPD) was the third-most common cause of death among the top 10 causes, accounting for about 5% of all deaths [126]. The current treatment regimen for acute exacerbation of COPD (AECOPD) can only bring about a change in patients from the acute exacerbation phase to the stable phase; however, this condition cannot be completely cured. Traditional Chinese medicines exert a unique therapeutic effect. Li et al. [7] conducted a randomised controlled trial to determine the clinical efficacy of SY to treat AECOPD. Their experimental results show the PAF receptor as a potential target for the treatment of AECOPD. SY injection can alleviate pulmonary hypertension in patients with AECOPD, effectively alleviate right ventricular failure, and alleviate myocardial ischemia and reduce WOB to some extent. SY intervention can significantly shorten the average length of stay of patients (*P*=0.006), reduce the average hospitalisation costs, and shorten the time of mechanical ventilation. Meanwhile, the use of SY instead of other drugs to treat AECOPD saves limited medical resources and is associated with good medical benefits and social welfare. However, their study has some limitations such as the limited representation of clinical samples. With respect to scientific research methods and sample inclusion, there is an obvious gap bridging multicentre, large sample, and triple-blind trials. There is also a gap with respect to international standards in medical technology and examination methods. Due to these limitations, no samples were collected for cytology or molecular biology studies. Thus, the effective molecular mechanism of SY in the treatment of AECOPD could not be determined. To summarise, researchers worldwide should focus on performing more randomised controlled trials to further evaluate the clinical value of SY.

Hepatocellular carcinoma (HCC) is a malignant tumour associated with a high rate of mortality worldwide that poses a serious threat to human life and health [127]. Chemotherapeutic drugs such as cisplatin inhibit DNA replication and damage cell membranes and are widely used to treat cancer; however, their side effects limit their use. Previous studies have shown that Chinese medicines, including Chinese herbal medicines such as safflower, lead to an enhancement of the anti-tumour effect when combined with cisplatin [128, 129]. Ma et al. [11] used the combination of cisplatin and HSYA in a mouse model of liver cancer and specifically studied the anti-tumour effect of HSYA on HCC and its impact on the tumour immune micro-environment. The study found that after HSYA therapy, the tumour cells decreased significantly. Optimal tumour growth-inhibiting effect was obtained at a concentration of 1.13 mg/kg HSYA. In a study in Central Asia, HSYA was found to lower the expression of *Foxp3* and *Rorγt* in tumour tissue in the spleen, enhance immunity in mice, and reduce liver tissue damage due to cisplatin chemotherapy, thereby regulating the tumour immune micro-environment and exerting an anti-cancer effect. However, the immune micro-environment of the body is complex and involves several immune factors. The aforementioned study lacks systematicity and comprehensiveness. Moreover, the influence of HSYA on other important immune factors needs further elucidation.

DN is a serious complication resulting from changes in the renal structure and function in patients with diabetes. The incidence of DN is increasing and is the main cause of death in patients with diabetes [130]. Wang et al. [2] showed that SY can treat DN-related diseases by regulating haemodynamics, oxidative stress, fibrosis, apoptosis, and hypolipaemia. At the same time, SY was found to reduce the urinary albumin excretion rate, increase blood glucose ratio and effectively improve other DN-related indicators. SY injection can be used alone or in combination with other traditional Western medicine to treat DN. Danhong injection (DHI) is composed of the aqueous extracts of *Salvia miltiorrhiza* and safflower, and the combination of these traditional Chinese medicines is mostly used to treat cardiovascular and cerebrovascular diseases [131]. DHI is significantly effective in treating atherosclerosis [132] and can reduce oxidative stress and plasma lipid levels [133, 134]. Guo et al. [135] established a model of HUVECs induced by H_2_O_2_ and proved that DHI and its components can effectively alleviate autophagy in these cells. When used alone or in combination with Western medicine, it significantly improves the overall efficacy and has lower toxicity. Studies by Wang and a few others were not multicentre, large-sample, high-quality clinical trials; thus, the clinical safety and efficacy of SY are yet to be established. Although previous studies have reported SY to be effective in the treatment of DN, its side effects are unclear. Additional rigorous studies and in-depth experimental design are required to determine the role of the drug in treating DN. Thus, future studies should focus on establishing comprehensive and effective schemes for the standardisation of randomised controlled trials.

SY and sodium chloride injection is a newly reported combination that has the effect of activating blood and blood and pain. The combination of metoprolol and SY injection has been reported to be effective in treating unstable coronary heart disease or angina pectoris by increasing serum TC, TG, and LDL-C levels and alleviating the symptoms of these conditions [136–140]. Wang et al. [141, 142] found that the Chinese patent Taohong Siwu keli (granules) significantly inhibited platelet aggregation when administered to SD rats with thrombotic cardiovascular and cerebrovascular diseases. Bitong Keli (Granule), processed and refined from 18 traditional Chinese medicines (including safflower, Pueraria radix, and bougainvillea), is formulated according to the basic theory and experience of traditional Chinese medicine. Owing to its definitive curative effect, Bitong Keli has long been used in clinical practice. Clinically, Bitong Keli is mainly used to treat cervical spondylosis, radicular pain, limb numbness, and restricted limb movement [143, 144]. Chitosan (CS) micro-spheres have been recently reported as a new pharmaceutical delivery system. CS can be used to achieve long-term effects by regulating the drug-release rate and protecting it from enzymatic degradation. This new technology has been used to synthesise slow-release HSYA-CS micro-spheres, which greatly reduce the number of medications, extend drug activity, and improve drug efficacy after injection. HSYA-CS can be prescribed at a lower dose and is more efficacious compared with traditional HSYA formulations [145].

## 5. Discussion

Although multiple pharmacological effects of SY have been reported, it continues to be a popular research field. One of the important reasons is that the new therapeutic effects that are being discovered by combining HSYA with other active components of traditional Chinese medicine show broad application prospects, and the mechanisms of action warrant elucidation at multiple levels. Other components of safflower are known to have extensive physiological activities and are of great value for further research. The complete research and development of safflower resources will be significant in guiding clinical practice and new drug development.

The application of SY in the medical field is extensive. SY has high stability and several pharmacological activities; however, its effects may be altered by light or exposure to the sun. Although several studies exist on the pharmacological activities of SY in recent years, some are relatively superficial and unsuitable to be used as a theoretical basis for further experimental research to promote the research and development of relevant drugs. This is one of the factors limiting the medicinal value of SY. As mentioned earlier, no samples were collected for cytology or molecular biology studies; thus, the effective molecular mechanism of SY in the treatment of AECOPD could not be determined. The protective mechanism of SY on myocardial I/R injury needs further study and experimental analysis. Some studies on SY lack systematicity and comprehensiveness. The clinical safety and efficacy are limited and the side effects of SY during the treatment of DN are still unclear.

Drugs can be developed according to clinical needs. The active components of SY can be extracted and separated, and their structures can be optimised to synthesise relevant drugs. SY exerts its effects by a combination of multiple pharmacological functions. It can be used alone and also combined with Western medicine to treat related diseases. Its toxicity is lower than that of most drugs. Future studies should be focused on the collaboration of domestic and foreign pharmaceutical researchers to conduct randomised controlled trials for an in-depth study on the mechanisms of action of the active ingredients of SY. This would enable more comprehensive and effective standardised treatment schemes to be introduced, help identify side effects, and shed light on drug safety, clinical applications, and precise specifications, thereby highlighting the clinical value of SY in promoting drug research and development and benefitting the society at large.

## 6. Conclusions

SY is a natural yellow pigment extracted from safflower petals. The major active substances of SY are water-soluble flavonoids. SY has multiple pharmacological activities, including alleviating cardiovascular and cerebrovascular diseases and abirritation. Moreover, it is known to have anti-cancer effects and is reported to be useful in treating diabetic complications. Therefore, SY is widely used in a clinical setting. Further research on the compatibility, stability, and thermal instability of SY is warranted. Compatibility and stability are crucial factors governing medicinal use. Thus, in-depth studies on the thermal instability of SY are significant prior to the development of SY-containing products.

## Figures and Tables

**Table 1:** The ingredients of safflower yellow.

No.	CAS number	Ingredients	Molecular formula	Structural formula
1	73-24-5	Adenine	C_5_H_5_N_5_	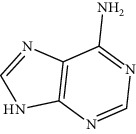

2	98-79-3	Pyroglutamic acid	C_5_H_7_NO_3_	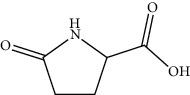

3	58-61-7	Adenosine	C_10_H_13_N_5_O_4_	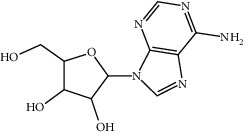

4	197227-95-5	Guanosine hydrate	C_10_H_13_N_5_O_5_	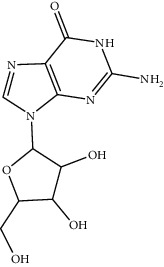

5	63-91-2	Phenylalanine	C_9_H_11_NO_2_	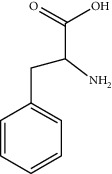

6	492-27-3	Kynurenic acid	C_10_H_7_NO_3_	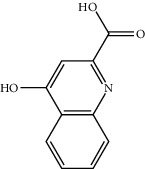

7	78281-02-4	Hydroxysafflor yellow A (HSYA)	C_27_H_32_O_16_	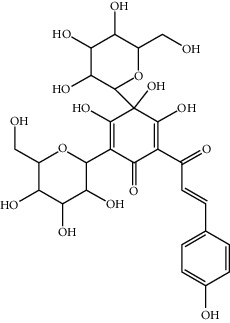

8	501-98-4	p-Coumaric acid	C_9_H_8_O_3_	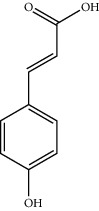

9	85532-77-0	Safflor yellow A	C_27_H_30_O_15_	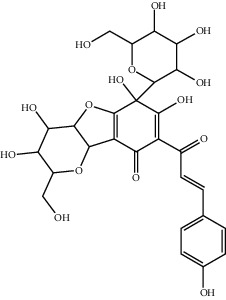

10	145134-62-9	6-Hydroxykaempferol-3,6,7-tri-O-*β*-glucoside	C_33_H_40_O_22_	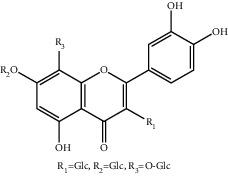

11	142674-16-6	6-Hydroxykaempferol-6,7-di-O-*β*-glucoside	C_27_H_30_O_17_	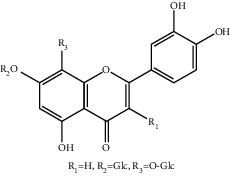

12	142674-16-6	6-Hydroxykaempferol-3,6-di-O-*β*-glucoside	C_27_H_30_O_17_	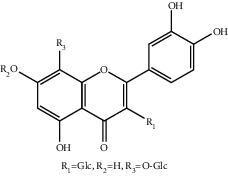

13	145134-63-0	6-Hydroxykaempferol-3-O-*β*-rutinoside-6-O-*β*-D-glucoside	C_33_H_40_O_21_	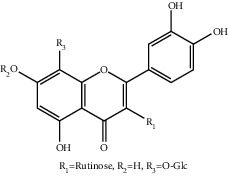

14	145134-61-8	6-Hydroxykaempferol-3-O-*β*-D-glucoside	C_21_H_20_O_12_	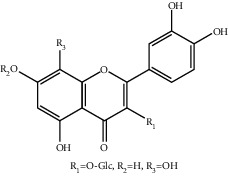

15	205527-00-0	6-Hydroxykaempferol-3-O-*β*-rutinoside	C_27_H_30_O_16_	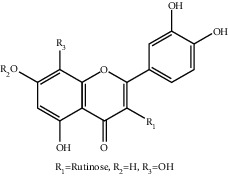

16	—	Hydroxycartormin	C_27_H_31_NO_14_	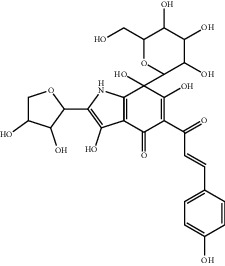

17	91574-92-4	Safflor yellow B (SYB)	C_48_H_54_O_27_	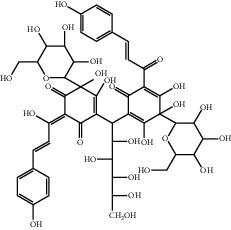

18	184840-84-4	Anhydrosafflor yellow B (AHSYB)	C_48_H_52_O_26_	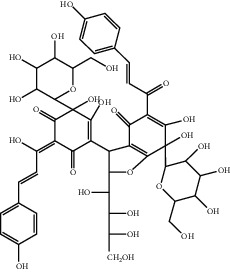

19	29741-09-1	Apigenin-7-O-*β*-glucuronic acid	C_21_H_18_O_11_	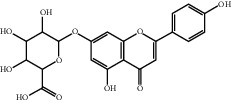

20	79974-25-7	Cartormin	C_27_H_29_NO_13_	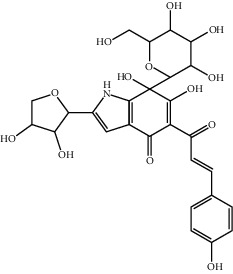

21	126093-98-9	Safflomin C	C_30_H_30_O_14_	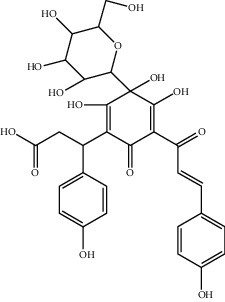

22	19895-95-5	Kaempferol-3-O-*β*-sophorose	C_27_H_30_O_16_	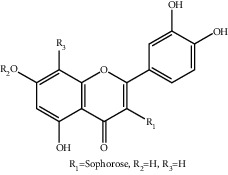

23	36535-79-2	Quercetin-3-O-*β*-rutinoside (Rutin)^a^	C_27_H_30_O_16_	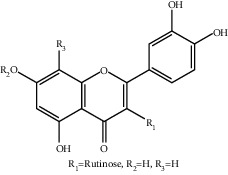

24	117-39-5	Quercetin^a^	C_15_H_10_O_7_	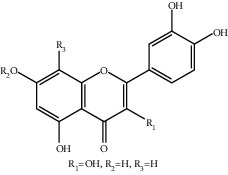

25	90327-16-5	Quercetin-3-O-*β*-glucoside^a^	C_21_H_20_O_12_	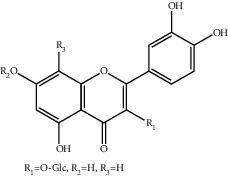

26	17650-84-9	Kaempferol-3-O-*β*-rutinoside	C_27_H_30_O_15_	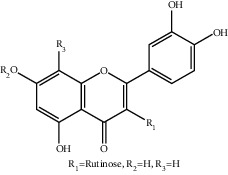

27	520-18-3	Kaempferol^a^	C_15_H_10_O_6_	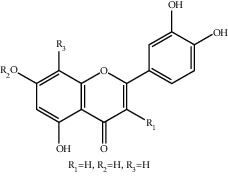

28	31159-41-8	Kaempferol-3-O-*β*-glucoside^a^	C_21_H_20_O_11_	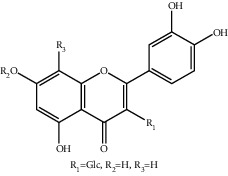

29	865688-88-6	Quercetin-3-*C*-*β*-glucoside	C_21_H_20_O_11_	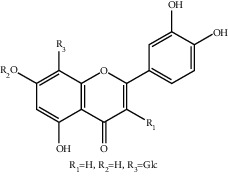

**Table 2:** The disease-resistant components and mechanism of safflower yellow (↑ increase and ↓ decrease).

Research compounds	Mechanisms	Models	Effect (dose)	Reference(s)
HSYA	↓Glutathione (GSH), malondialdehyde (MDA)	Brain injury rats	Anti-craniocerebral injury (10 g/kg)	[14]
	↑SOD activity			
	↓Cytochrome C, caspase-3, reactive oxygen species (ROS), Mst1/caspase-3 signal pathway	H9c2 cell and Sprague-Dawley (SD) rats cardiomyocytes cells	Protective effects of hypoxia/reoxygenation on myocardial injury (20 *μ*mol/L)	[15]
	↑Akt and HKII			
	↑mRNA expression of p53 gene	Human umbilical vein endothelial cell (EC-304)	Inhibition of angiogenesis (0.33 mg/L)	[16]
	↓mRNA expression of c-myc, VEGF, bFGF, HSPG			
	↓mRNA expression of IL-6, IL-10, TNF-*α*	Septic rats	Anti-inflammatory (120 mg/kg)	[17]
	↓CD4^+^CD25^+^FOXP3^+^ Tregs/CD4^+^T lymphocytes	Liver cancer rats	Anti-cancer (1.13 mg/kg)	[18]
	↓ROS, BAX, PTEN, TLR4, MyD88, NF-*κ*B mRNA, NF-*κ*B (p65)	Human umbilical vein endothelial cells (HUVECs)	Anti-oxidation (8 *μ*g/mL)	[19]
	↑GSH, AKT, SOD, Bcl-2			
	↓LOX-1 mRNA, LDH	Human coronary artery endothelial cells (HCAECs)	Anti-oxidation (0.2 mM)	[20]
	↑eNOS, NO			
	↓NLRP3, ASC, caspase-1, GSDMD, IL-1*β*, IL-18, LDH, NF-*κ*B, p-p56	Cerebral ischemia-reperfusion injury (CIRI)	Neuroprotective (20 *μ*M)	[21]
	↑NO content	Leukaemia cells in rat macrophage (RAW 264.7)	Analgesic (0.1 mmol/L)	[22, 23]
	↑MAPK/p38/iNOS signal pathway			
	↓Contents of IL-6, TNF-*α*, IL-1*β*; MLC phosphorylation, inflammation due to PAF	Human bronchial smooth muscle cells (HBSMCs)	Anti-inflammatory (81 *μ*mol/L)	[24]
	↓Contents of IL-1*β*, IL-6, TNF-*α*, COX-2, iNOS; Bcl-2/Bax ratio	Coronary heart disease male miniature pigs	Anti-inflammatory (40 mg/kg)	[25]
	↑Phosphorylation of the JAK2/STAT3 pathway			
	↓Micro-vessel density (MVD) in BGC-823 tumour tissue	Human gastric adenoma nude rats	Anti-tumour (0.028 g/L)	[26]
	↓Fatty acid synthetase (FASN), peroxisome proliferator-activated receptor-gamma (PPAR-*γ*), stearoyl-CoAdesaturase1(SCD1)	Hyperlipidaemia and fatty liver rats	Regulation of fatty liver (30 mg/kg)	[27]
	↓Matrix metalloproteinase-2 (MMP2)	Human breast cancer cells (MDA-MB-231)	Anti-tumour (1 mM)	[28]
	↓TNF-*α*, ICAM-1, IL-1*β*, IL-6	Acute lung injury rats	Alleviating inflammatory response in the lungs (750 mg/kg)	[29]
	↓TNF-*α*, IL-6	Human bone articular chondrocytes	Anti-osteoarthritis (20 mg/L)	[30]
	↑miR-140-5p			
	↓IL-1*β*, PTGS2, MMP-13	Rat chondrocytes	Anti-osteoarthritis (10 *μ*M)	[31]
	↑COL2A1, ACAN			
	↓N-cadherin proteins, vimentin proteins, ROS	Ovarian cancer SKOV-3 cells	Anti-tumour (0.25 mg/L)	[32]
	↓E-cadherin protein			
SYB	↓2-deoxyribose oxidation	Fenton reaction in vitro	Anti-oxidation (2 mmol/L)	[33]
	↑Nuclear factor erythrocyte related factor 2 (Nrf2), heme oxygenase 1 (HO-1), NAD(P)H dehydrogenase	Human hepatocellular carcinoma cells (HepG2)	Anti-oxidation (150 nmol/L)	[6]
	↑Serum neuron specific enolase (NSE), human S100B (S-100B)	Brain ischemia/reperfusion (I/R) rats	Anti-inflammatory (8 mg/kg)	[34]
Quercetin	↓ROS, caspase-3	Steoarthritis (OA) rats	Anti-inflammatory (8 *μ*M)	[35]
	↑TGF-*β*1, TGF-*β*2 in synovia			
Kaempferol	↓p-mTOR, p-PI3K, p-AKT protein	Endometrial cancer cells (MFE-280)	Anti-cancer (10 *μ*M)	[36]
p-Coumaric acid	↓Blood glucose, creatinine, blood urea nitrogen (BUN), MDA, urine protein (PRO)	Diabetic nephropathy rats	Anti-inflammatory, anti-oxidation (100 mg/kg)	[37]
	↑SOD activity			
Kynurenic acid	↑Heme oxygenase-1 (HO)-1	Human umbilical vascular endothelial cells (HUVECs)	Anti-inflammatory (100 *μ*M)	[38]
	↓HUVECs inflammation, phosphorylation of NF-*κ*B			

**Table 3:** Medicines containing safflower yellow.

NO.	Product name	Major components	Production enterprise	Function	URL
1	The invention relates to a safflower injection rich in HSYA and its preparation	HSYA	Lanzhi Group Wanrong Pharmaceutical Co. Ltd.	Promote blood circulation and remove blood stasis, increase coronary flow and myocardial nutrient blood flow, effectively dilate blood vessels, inhibit thrombosis and platelet aggregation, and reduces inflammation	https://pss-system.cnipa.gov.cn/sipopublicsearch/patentsearch/showViewList-jumpToView.shtml

2	The use of HSYA during the preparation of health drugs or functional foods for the treatment of hypoxic pulmonary hypertension	HSYA	Harbin Medical University	Prevent and treat pulmonary hypertension	https://pss-system.cnipa.gov.cn/sipopublicsearch/patentsearch/showViewList-jumpToView.shtml

3	A drug composition for the treatment of kidney diseases and its pharmaceutical use	Rhubarb acid, emodin methyl ether, emodin glycoside, rhubarb glycoside, danshensu, salvianolic acid B, HSYA, astragaloside iv, astragalus polysaccharide, salvianolic acid A, and mullein isoflavone glycoside	Xi' An Cntury Shengkang Pharmaceutical Co. Ltd.	For the treatment of kidney disease, renal failure, nephrotic syndrome, cardiovascular and cerebrovascular diseases, and tumours	https://pss-system.cnipa.gov.cn/sipopublicsearch/patentsearch/showViewList-jumpToView.shtml

4	Application of safflower yellow during the preparation of drugs for treatment and/or prevention of acute soft tissue injury	Safflower yellow	Beijing Institute of Cardiopulmonary and Vascular Diseases	Treat and/or prevent acute soft tissue injury caused by inflammatory factors	https://pss-system.cnipa.gov.cn/sipopublicsearch/patentsearch/showViewList-jumpToView.shtml

5	Compound combinations, with peach red siwu soup as the source	Peach kernel, safflower (safflower yellow), angelica sinensis, rehmannia glutinosa, white peony root, and six chuanxiong herbs	Jinan University	Prevent and cure cardiovascular diseases	https://pss-system.cnipa.gov.cn/sipopublicsearch/patentsearch/showViewList-jumpToView.shtml

6	Application and dehydration of safflower yellow B during the manufacture of gastric cancer drugs	AHSYB	Binzhou Medical College	As an ERK regulator to cell cycle to inhibit gastric cancer cell proliferation	https://pss-system.cnipa.gov.cn/sipopublicsearch/patentsearch/showViewList-jumpToView.shtml

7	The utility model related to a drug composition for the treatment of cardiovascular and cerebrovascular diseases	Salvianolic acid B and HSYA were the main active components	Chengdu Purifa Drug Development Co. Ltd.	Prevent or treat cardiovascular and cerebrovascular diseases	https://pss-system.cnipa.gov.cn/sipopublicsearch/patentsearch/showViewList-jumpToView.shtml

8	Application of HSYA during the preparation of drugs for treating sepsis	HSYA	Tianjin Chase Sun Pharmaceutical Co. Ltd.	Treat sepsis	https://pss-system.cnipa.gov.cn/sipopublicsearch/patentsearch/showViewList-jumpToView.shtml

9	The invention relates to the procyanidin safflower yellow compound soft capsule and its preparation	These include safflower seed oil, procyanidins, safflower yellow, sodium alginate, and Tween-80	Urumqi Shangshanyuan Biotechnology Co. Ltd.	Prevent or treat cardiovascular and cerebrovascular diseases; improve memory, slow aging, prevent stroke, promote cholesterol decomposition, moisturise the skin, and maintain health	https://pss-system.cnipa.gov.cn/sipopublicsearch/patentsearch/showViewList-jumpToView.shtml

10	The invention relates to shu jin huo xue capsules and their preparation	Safflower (safflower yellow), rhizoma cyperi (system), and dog ridge (system)	Hangzhou East China Pharmaceutical Group Kangrun Pharmaceutical Co. Ltd.	Relax tendons	https://pss-system.cnipa.gov.cn/sipopublicsearch/patentsearch/showViewList-jumpToView.shtml

11	The invention relates to a fingerprint of shu jin huo xue preparation and its application in overall quality evaluation	Safflower (safflower yellow), rhizoma corydalis (made), dog ridge (made), xiangjia skin, mistletoe, zeilanthus leaves, xiejin grass, luoshi rattan, caulis spatholobus, natural copper (forged), and ten kinds of single medicines	Zhejiang University of Technology	Used for the pain of muscles and bones, fall injury, rheumatoid arthritis, and other diseases	https://pss-system.cnipa.gov.cn/sipopublicsearch/patentsearch/showViewList-jumpToView.shtml

12	The invention relates to a natural composition that can improve the disturbance of blood micro-circulation and osteoporosis and the preparation method	It uses *Panax notoginseng*, peach kernel, bone scissora, safflower (safflower yellow), angelica sinensis, maca, and licorice as raw materials	Ezhou Institute of Industrial Technology, Huazhong University of Science and Technology; Huazhong University of Science and Technology	Improve micro-circulation in bone; promote oxygen, calcium, and other nutrients to enter the bone; and encourage bone metabolism to return to normal, so as to effectively improve osteoporosis	https://pss-system.cnipa.gov.cn/sipopublicsearch/patentsearch/showViewList-jumpToView.shtml

13	Application of dehydrated safflower yellow B during the preparation of breast cancer drugs	AHSYB	Binzhou Medical College	Inhibit the proliferation of breast cancer cells	https://pss-system.cnipa.gov.cn/sipopublicsearch/patentsearch/showViewList-jumpToView.shtml

14	The invention relates to a composition containing type II collagen and a preparation process thereof	Type II collagen, turmeric, active protectant, and colourant (safflower yellow)	Beijing Suwei Biological Technology Co. Ltd.	Inhibit inflammatory response, improve the role of arthritis, and assist collagen treatment of inflammatory diseases	https://pss-system.cnipa.gov.cn/sipopublicsearch/patentsearch/showViewList-jumpToView.shtml

15	The invention relates to a traditional Chinese medicine composition for treating scars and its preparation and application	Danshen extract, chuanxiong extract, matrine extract, gallnut extract, safflower extract (safflower yellow), brucea javanica extract, and excipients	Guizhou University of Traditional Chinese Medicine	Cure scars	https://pss-system.cnipa.gov.cn/sipopublicsearch/patentsearch/showViewList-jumpToView.shtml

16	Application of HSYA in the preparation of tinnitus drugs and drug boxes	HSYA	Binzhou Medical College	Treat anxiety and depression caused by tinnitus	https://pss-system.cnipa.gov.cn/sipopublicsearch/patentsearch/showViewList-jumpToView.shtml

## Data Availability

Data sharing is not applicable to this article as no data sets were generated or analysed during the current study.

## Abbreviations

SY: Safflower yellow
HSYA: Hydroxysafflor yellow A
HSYB: Hydroxyl safflower yellow pigment B
AHSYB: Anhydrosafflor yellow B
CCVDs: Cardiovascular and cerebrovascular diseases
ASO: Atherosclerosis
HUVECs: Human umbilical vein endothelial cells
EC: Endothelial cells
GSH: Glutathione
COPD: Chronic obstructive pulmonary disease
AECOPD: Acute exacerbation of chronic obstructive pulmonary disease
MDA: Malondialdehyde
GLU: Glutamic acid
SOD: Superoxide dismutase
HG: High glucose
HCAECs: Human coronary artery endothelial cells
OX-LDL: Oxidised low-density lipoprotein
eNOS: Endothelial nitric oxide synthase
LOX-1: Low-density lipoprotein receptor 1
LDH: Lactate dehydrogenase
ICAM-1: Intercellular adhesion molecule-1
VCAM-1: Vascular cell adhesion molecule-1
FIB: Fibrinogen
PT: Prothrombin time
PAF: Platelet-activating factor
PAI: Plasminogen activator
t-PA: Tissue plasminogen activator
AT-III: Anti-thrombin III
MI/R: Myocardial ischemia/reperfusion
MI: Myocardial infarction
I/R: Ischemia/reperfusion
A/R: Hypoxia/reoxygenation
PI3K/Akt/GSK3*β*: Phosphatidylinositol-3-kinase/protein kinase B/glycogen synthase kinase 3*β*
Ang II: Angiotensin II
TXB_2_: Thromboxane B_2_
OGD: Oxygen-glucose deprivation
BMECs: Brain micro-vascular endothelial cells
AS-IV: Astragaloside IV
CIRI: Cerebral ischemia-reperfusion injury
CAT: Catalase
NSE: Neurone specific enolase
S-100B: S-100B protein
MVD: Micro-vessel density
CRE: Creatinine
BUN: Blood urea nitrogen
PRO: Proline
Akt: Protein kinase B
HKII: Hexokinase
ROS: Reactive oxygen species
Caspase-3: Cysteine aspartic protease
Mst1: Protein kinase MST
Cyt-c-: Cytochrome C
HETE: 15-hydroxyeicosatetraenoic acid
BBB: Blood-brain barrier
mirna-1: Micro-RNA-1
TLR4: Toll-like receptor 4
NF-*κ*B p65: Nuclear transcription factor-*κ*B
TNF-*α*: Tumour necrosis factor-*α*
IL-6: Interleukin-6
MAPK: Mitogen-activated protein kinase
ALI: Acute lung injury
LPS: Lipopolysaccharide
LDL-C: Low-density lipoprotein
TG: Triglyceride
TC: Total cholesterol
HDL-C: High-density lipoprotein
FASN: Fatty acids synthase
PPAR-*γ*: Peroxisome proliferator-activated receptor
SCD1: Stearoyl-coadesaturase1
Sirt1: Silent information regulator1
TN: Triple-negative
MMP2: Matrix metalloproteinase-2
EGF: Epidermal growth factor
HCC: Hepatocellular carcinoma
DN: Diabetic nephropathy
DM: Diabetes mellitus
WHO: World Health Organization
UAER: Urinary albumin excretion rate
DHI: Danhong injection
CS: Chitosan.
